# P-1725. A Cluster Randomized Trial of Bundled Interventions to Reduce Inappropriate Antibiotic Use in the Outpatient Setting

**DOI:** 10.1093/ofid/ofae631.1889

**Published:** 2025-01-29

**Authors:** Sherin Shams, Asma Ali Al Nuaimi, Jeyaram Illiayaraja Krishnan, Samah Saleem, Maryan Hassan Aziz, Naheel Ismail Seyam, Kamran Aziz, Dalia Kandil, Aimon Malik, Anil G Thomas, Hanaa Nafady-Hego, Wael Ezzeldin Saeed, Sandy Semaan, Samya Ahmad Al Abdulla, Mohamed Ghaith Al-Kuwari, Abdul-Badi Abou-Samra, Adeel A Butt

**Affiliations:** Hamad Medical Corporation, Doha, Ad Dawhah, Qatar; Primary Health Care Corporation, Doha, Qatar, Doha, Ad Dawhah, Qatar; Primary Health Care Corporation, Doha, Qatar, Doha, Ad Dawhah, Qatar; Hamad Medical corporation, Doha, Ad Dawhah, Qatar; Primary Health Care Corporation, Doha, Qatar, Doha, Ad Dawhah, Qatar; Primary Health Care Corporation, Doha, Qatar, Doha, Ad Dawhah, Qatar; Primary Health Care Corporation, Doha, Qatar, Doha, Ad Dawhah, Qatar; Primary Health Care Corporation, Doha, Qatar, Doha, Ad Dawhah, Qatar; Hamad Medical corporation, Doha, Ad Dawhah, Qatar; Hamad Medical corporation, Doha, Ad Dawhah, Qatar; Microbiology and immunology, Faculty of Medicine, Assiut University, Assiut, Egypt., Doha, Ad Dawhah, Qatar; Primary Health Care Corporation, Doha, Qatar, Doha, Ad Dawhah, Qatar; Primary Health Care Corporation, Doha, Qatar, Doha, Ad Dawhah, Qatar; Primary Health Care Corporation, Doha, Qatar, Doha, Ad Dawhah, Qatar; Primary Health Care Corporation, Doha, Ad Dawhah, Qatar; Hamad Medical corporation, Doha, Ad Dawhah, Qatar; Weill Cornell Medicine, Doha, Ad Dawhah, Qatar

## Abstract

**Background:**

Antibiotics are often inappropriately prescribed in the outpatient settings. Among the most common reasons for inappropriate antibiotic prescriptions are upper respiratory tract infections (URI) which are known or highly likely to be viral in origin. Our aim was to test a bundle of complementary interventions to reduce inappropriate antibiotic prescriptions in the outpatient setting for patients presenting with various URIs. Here we present preliminary data from the cluster randomized trial using pre-intervention baseline data (August to October 2023) and after implementation of interventions (December 2023 to March 2024).

**Figure 1.**
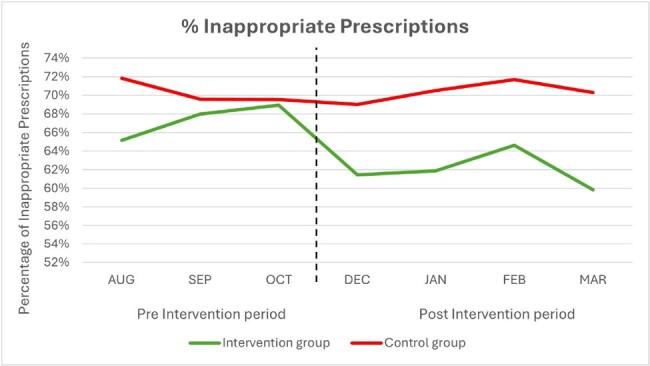
Comparison of inappropriate antibiotic prescriptions for URTIs between the intervention and control groups during the pre-intervention (Aug 2023 – Oct 2023) and post-intervention phases (Dec 2023 - Mar 2024).

**Methods:**

We randomly assigned 2 Primary Healthcare Centers (PHCC) in Qatar to receive a 4 component intervention: 1) extensive provider education; 2) providing an algorithm driven decision support tool based on professional society recommendations for appropriate use of antibiotics for various URIs; 3) option for deferred prescription; and 4) monthly feedback to all physicians regarding their prescription patterns including anonymized comparison with their peers. Two PHCCs in the control group received a single intervention which was randomly chosen. For this study, we identified 4 common URIs for which professional society guidelines do not recommend use of antibiotics (acute bronchitis, acute laryngitis, common cold, influenza without mention of pneumonia). Prescriptions written and corresponding diagnoses were retrieved from the electronic medical records. Children < 2 years of age, and individuals with any immune compromising condition or medications were excluded.

**Table. 1:**
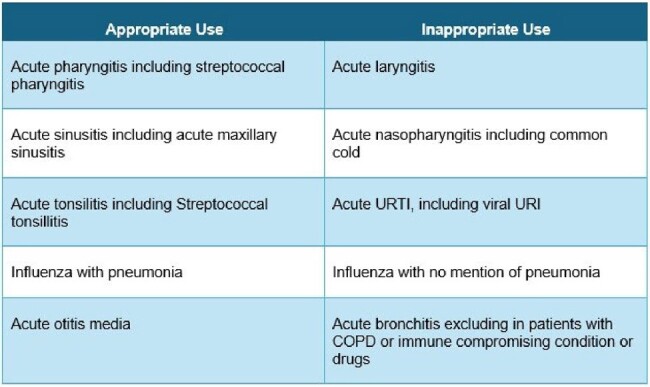
Appropriate and Inappropriate use of antibiotics in URTIs

**Results:**

A total of 7,246 antibiotic prescriptions were written for 4 included URIs during the study period. The percentage of inappropriate prescriptions in the intervention group decreased from 67.7% to 62% in the first 4 months after the intervention (p-value >0.05). The proportion of inappropriate prescriptions in the control groups remained steady at 70% before and after the intervention.

**Conclusion:**

While not statistically significant in this early stage, our preliminary results indicate a trend in reduction in inappropriate prescriptions of antibiotics for URIS in the outpatient setting.

**Disclosures:**

**Adeel A. Butt, MD, MS**, Gilead Sciences: Grant/Research Support

